# Expression, activity and localization of lysosomal sulfatases in Chronic Obstructive Pulmonary Disease

**DOI:** 10.1038/s41598-018-37958-w

**Published:** 2019-02-13

**Authors:** Julie Weidner, Prajakta Jogdand, Linnea Jarenbäck, Ida Åberg, Dalja Helihel, Jaro Ankerst, Gunilla Westergren-Thorsson, Leif Bjermer, Jonas S. Erjefält, Ellen Tufvesson

**Affiliations:** 1 Respiratory Medicine and Allergology, Department of Clinical Sciences Lund, Lund University, Lund, Sweden; 2 Airway Inflammation, Department of Experimental Medical Science, Lund University, Lund, Sweden; 3 Lung Biology, Department of Experimental Medical Science, Lund University, Lund, Sweden

## Abstract

Chronic obstructive pulmonary disease (COPD) is a leading cause of death world-wide. Recently, we showed that COPD is associated with gene polymorphisms in *SUMF1*, a master regulator of sulfatases. Sulfatases are involved in extracellular matrix remodeling and activated by SUMF1, but their role in the lung is poorly described. We aimed to examine how sulfatases are affected in the airways of patients with COPD compared to ever smokers and never smokers. We observed that mRNA expression of the sulfatases *GALNS*, *GNS* and *IDS* was increased, while protein expression of many sulfatases was decreased in COPD fibroblasts. Several sulfatases, including GALNS, IDS, and SGSH, showed increased activity in COPD fibroblasts. Examination of different sulfatases by immunofluorescence showed that IDS, ARSB, GNS and SGSH in fibroblasts were localized to sites other than their reported destination. Using a master panel from different organs, RNA expression of all sulfatases could be observed in lung tissue. Additionally, immunohistochemistry on lung biopsies indicated differing expression of sulfatases in COPD patients. In conclusion, mRNA, protein expression, sulfatase activity levels, and localization of sulfatases are altered in lung fibroblasts and lung tissue from COPD patients and may be mechanistically important in COPD pathogenesis. This could contribute to the understanding of the disease mechanism in COPD and in the long run, to lead to more individualized therapies.

## Introduction

Chronic obstructive pulmonary disease (COPD) has become one of the main causes of morbidity and mortality worldwide^1^. It is a heterogeneous disease which includes thickening of the small airways and destruction of the alveoli, leading to emphysema^2^. One of the main causes of COPD is cigarette smoking, though other environmental and genetic factors^3^ can lead to development of the disease^4^. Although much is known about the development of COPD, there is currently no treatment to cure the disease. Furthermore, the molecular and cellular changes that occur in the lungs of COPD patients versus ever smokers and never smokers remain to be fully explored.

The sulfatase modifying factor, SUMF1, is the highly conserved master regulator of all sulfatases in eukaryotic cells^5^. There are 17 known human sulfatases which are all activated by SUMF1 through the alteration of a conserved cysteine residue to a c-alpha formylglycine^6,7^. Sulfatases are critical molecules that act to modify proteoglycan chains through the removal of sulfate groups^8,9^, thereby changing the characteristics of these molecules^10^. In this study, we focused on five sulfatases (ARSB, GNS, GALNS, IDS and SGSH) which are known to be involved in desulfation of glycosaminoglycan chains on proteoglycans^5,6^. Glycosaminoglycans are important components in extra cellular matrix turnover in the lungs^11^, and the altered extra cellular matrix composition is an important factor in COPD^12^. ARSA, -G and -K are also categorized as lysosomal sulfatases^6,13,14^, but the role of these sulfatases in extracellular matrix remodeling is not apparent, especially not in the context of COPD. Recent studies have observed a link between the destruction of alveoli and *SUMF1*^15,16^. *SUMF1* deficient mice (*Sumf1*−/−*)* developed a lung phenotype similar to the emphysema that is seen in humans. Additionally, these mice accumulated sulfated glycosaminoglycans, which had an inhibitory effect on the alveolarization of the lung.

Several lysosomal storage disorders have already been attributed to lack of, or alterations in, various sulfatases^17–19^. Knockout mice for several of the sulfatases have been created and are giving insight into the effects that changes to sulfatase levels have in the body^5,6,20–22^. These diseases often manifest during childhood and, thus far, the only known treatments are through bone marrow transplant and gene therapy^22,23^. Examples of diseases caused by deficiency of sulfatases include Mucopolysaccaridosis II (Hunter’s syndrome^24^) and Mucopolysaccharidosis III (Sanfilippo Syndrome^25^), which present developmental delays, developmental regression and a much shorter life expectancy.

Recently, we have reported that polymorphism in the *SUMF1* gene is associated with having COPD and that several single nucleotide polymorphisms (SNPs) in *SUMF1* affect mRNA expression of *SUMF1*^26^. This association of the *SUMF1* gene with COPD, led us to question whether the sulfatases, which are directly influenced by SUMF1, were also affected in COPD. Additionally, little is known about the expression of sulfatases and their role in the human lung or in the context of COPD. Therefore, we aimed to examine a subgroup of sulfatases, the lysosomal sulfatases involved in desulfation of glycosaminoglycans, and to characterize their role in COPD.

## Results

### Sulfatase mRNA levels

**Figure 1:**
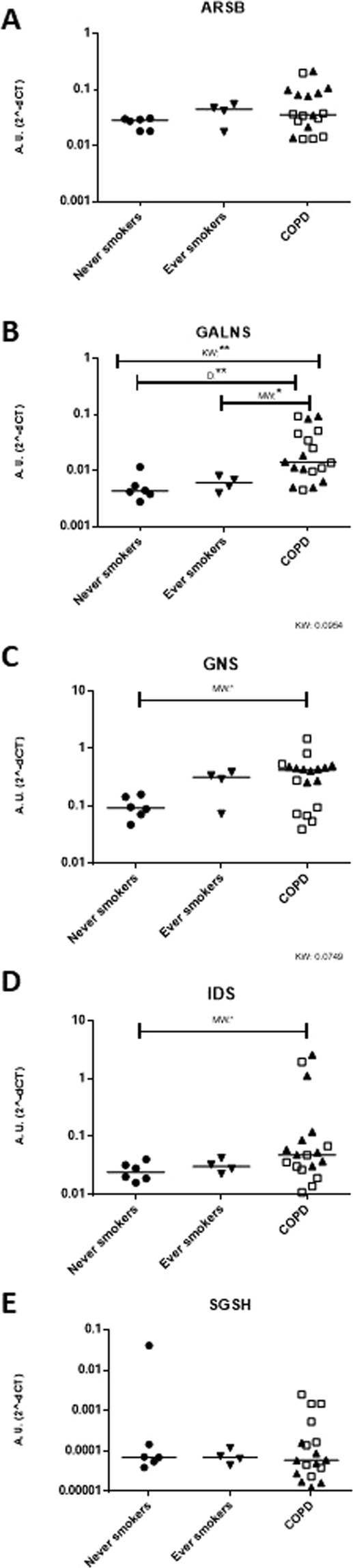
mRNA expression of lysosomal sulfatases is increased in COPD patients. mRNA expression of *ARSB* (**A**), *GALNS* (**B**), *GNS* (**C**), *IDS* (**D**), and *SGSH* (**E**) were observed in lung fibroblasts from never smokers, ever smokers and COPD patients. Line indicates the median value. Former smokers are represented as filled triangles and current smokers are represented by open squares. K-W = Kruskal-Wallis test was used, followed by Dunn’s multiple comparison post-tests (=D). In some cases, relationships were also determined by Mann-Whitney test = M.W. **= significance at p < 0.01, *= significance at p < 0.05. Data are depicted as 2^−∆Ct^ showing the expression of the respective specific mRNA normalized against the average expression of the two reference genes *β-actin* and *GAPDH*. A.U. = arbitrary units.

We set out to ask if mRNA levels of sulfatases, the downstream targets of SUMF1, were affected in COPD patients. When comparing mRNA expression in lung fibroblasts from never-smokers, ever smokers, and COPD patients, we found that there was a significant increase in mRNA expression from COPD patients in *GALNS*, *GNS* and *IDS* mRNA (Fig. 1). Furthermore, amongst COPD patients, there was some clustering of subjects based on current smoking status, which was most apparent in *ARSB* and *GNS* expression, but was not significant.

### Lysosomal sulfatase activity is altered in COPD

**Figure 2:**
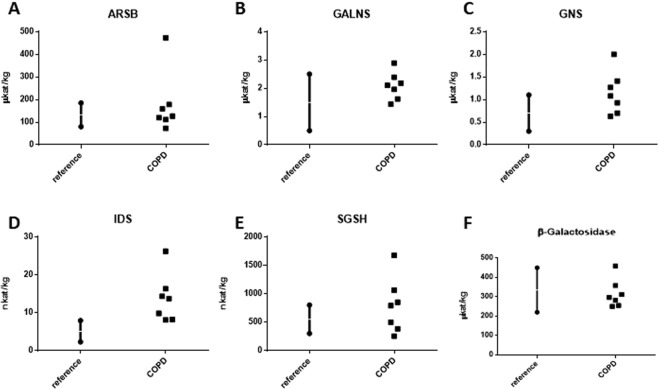
Sulfatase activity is increased in some COPD patients. The activity of the five lysosomal sulfatases: ARSB (**A**), GALNS (**B**), GNS (**C**), IDS (**D**), and SGSH (**E**) as well as a control, beta-galactosidase (**F**), was examined in cultured lung fibroblasts from COPD patients. Data are depicted as individual values and reference range depicts the range clinically used as normal range for healthy subjects.

As the mRNA levels of certain sulfatases were altered in COPD patients, we next asked if activity levels of these sulfatases were affected in lung fibroblasts. The activity levels of the lysosomal sulfatases: ARSB, GALNS, GNS, IDS, and SGSH were therefore measured (Fig. 2). We found that in all COPD patients examined, IDS activity was increased above the reference range (Fig. 2D), and in some, but not all, COPD patients we also observed an increase of GNS and SGSH activity (Fig. 2C and E). The ARSB and GALNS activity were within the reference range in the majority of patients (Fig. 2A,B). In all COPD subjects, the activity of the reference enzyme, beta-galactosidase, was within the reference range (Fig. 2F)

### Lowered sulfatase protein levels in COPD patients

**Figure 3:**
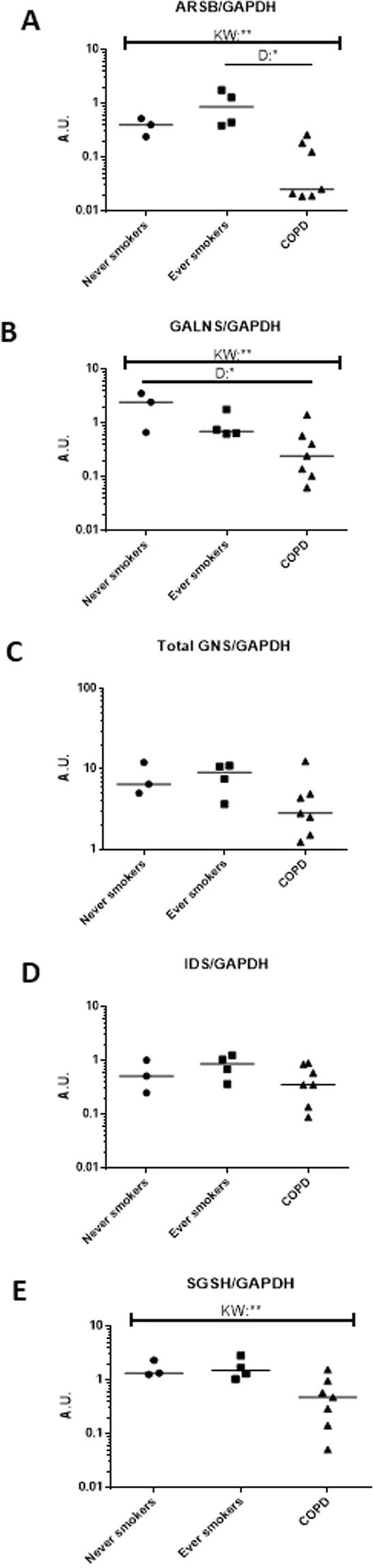
Protein expression of lysosomal sulfatases is decreased in COPD patients. Quantification of sulfatase expression (ARSB (**A**), GALNS (**B**), GNS (**C**), IDS (**D**) and SGSH (**E**)) versus GAPDH (control) from western blotting are shown in never smokers, ever smokers and COPD patients (**A**–**E**). GNS is comprised of a full length form and two bands formed through internal cleavage. Quantification for GNS was performed using all three defined bands. Each dot represents a different subject. **= significance at p < 0.01, *= significance at p < 0.05. A.U. = arbitrary units. K-W = Kruskal-Wallis test was used, followed by Dunn’s multiple comparison post-tests (=D).

Next we wanted to determine if the changes in mRNA expression and sulfatase activity levels were reflected at the protein level. Interestingly, we found that compared to never smokers and ever smokers, COPD patients showed a decrease in protein levels (Fig. S1). This change in protein expression was also evident when intensities of the bands were quantified (Fig. 3A–E).

### Localization of sulfatases in lung fibroblasts

**Figure 4:**
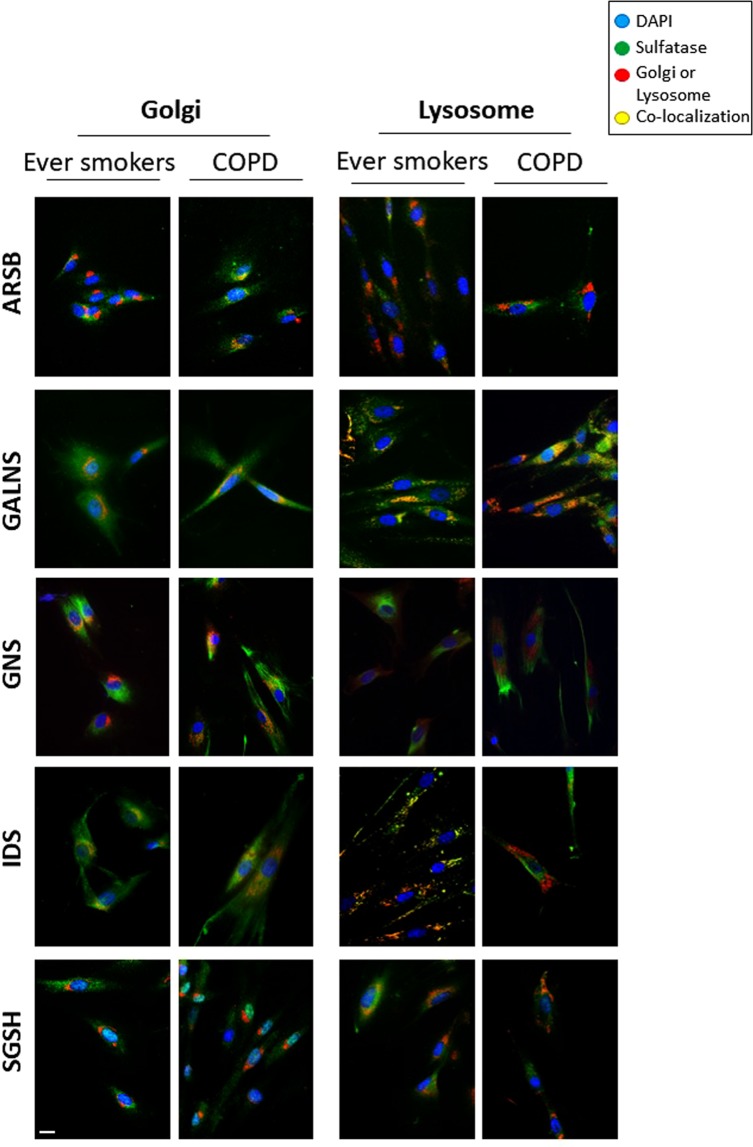
Localization of sulfatases in central airway fibroblasts. The sulfatases (ARSB, GALNS, GNS, IDS and SGSH) were examined for localization within cultured lung fibroblasts from ever smokers and COPD patients. Panels shown are representative images of at least three ever smokers and at least three COPD patients. Sulfatases are represented in green. Golgi (stained for Golgin97, left panels) or lysosomes (stained for LAMP1, right panels) are shown in red. Nuclei are shown in blue (DAPI). Yellow indicates co-localization of sulfatase and compartment. Scale bar equals 20 μm.

Due to changes in expression and activity levels of lysosomal sulfatases in cells from COPD patients, we aimed to examine their respective localization within the cells. ARSB, GALNS, GNS, IDS, and SGSH were stained in conjunction with marker proteins for the Golgi or lysosomes (Fig. 4). GALNS indeed showed a lysosomal localization in both ever smokers and COPD patients. IDS, on the other hand, was found to localize as expected to lysosomes^5^ in ever smokers, but not in COPD patients. Additionally, IDS appeared to partially co-localize with the Golgi in both ever smoker and COPD fibroblasts. SGSH appeared to partially co-localize to the lysosomes in ever smoker and COPD fibroblasts. GNS did not appear to co-localize with the Golgi or lysosomes, but instead showed a more reticulated structure throughout the cells in both ever smokers and COPD patients. Interestingly, in COPD fibroblasts, lysosomes appeared more perinuclear and the GNS staining more towards the periphery of the cell whereas in ever smokers the opposite pattern- GNS perinuclear and lysosomes closer to the periphery-was observed. Lastly, ARSB was found to localize to the Golgi in COPD fibroblasts, while its localization in ever smokers remained elusive.

### Sulfatase mRNA expression in the lung

**Figure 5:**
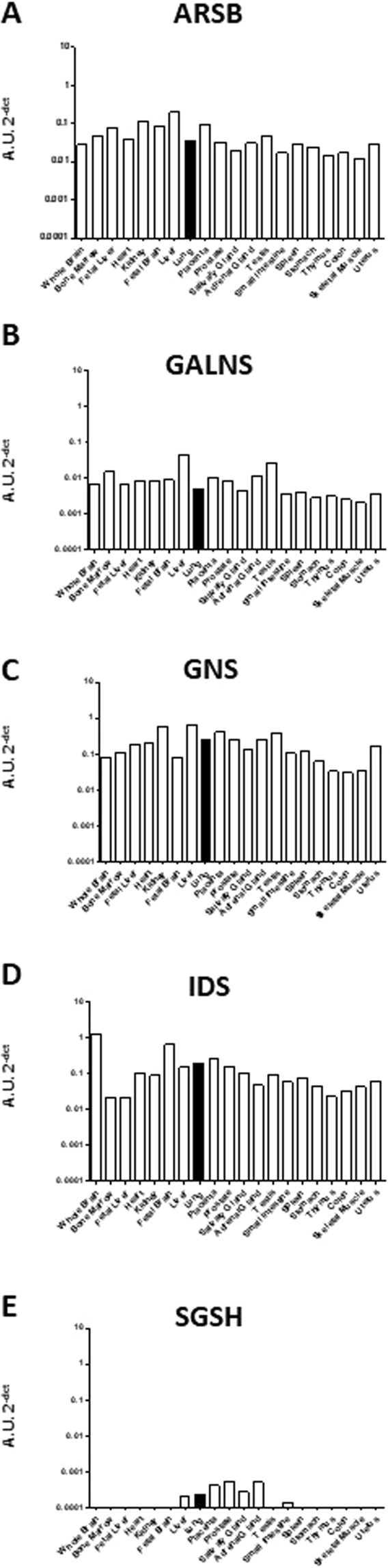
Lysosomal sulfatases are differentially expressed in whole lung tissue. Expression of the lysosomal sulfatases: *ARSB* (**A**), *GALNS* (**B**), *GNS* (**C**), *IDS* (**D**) and *SGSH* (**E**) using RNA from different body tissues were explored via a Human Total Master panel II RNA kit. The black bar indicates mRNA expression from whole lung. Data are depicted as 2^−∆Ct^ showing the expression of all specific mRNA normalized against the average expression of the two reference genes *β-actin* and *GAPDH*. A.U. = arbitrary units.

In addition to lung fibroblasts, sulfatase RNA expression in total lung tissue was investigated using the Human Total RNA Masterpanel Kit that includes 20 different tissues in the body. All sulfatases examined were found to be expressed in whole lung tissue (Figs 5 and S2). The five examined lysosomal sulfatases previously investigated in this study were all expressed in lung tissue, and specifically *GNS*, *IDS* and *SGSH* showed relatively high levels in lung tissue compared to other body tissues (Fig. 5A–E). *GNS* and *IDS* also showed the highest expression levels among the lysosomal sulfatases in whole lung tissue (Fig. 5B,C), and *SGSH* showed lowest levels (Fig. 5E), although detectable levels were still present. Of the other (non-lysosomal) sulfatases examined, *ARSJ* showed the highest expression in the lung compared to all other tissues and *SULF2* was the most highly expressed sulfatase of the 17 examined (Fig. S2).

### Localization of sulfatases in lung tissue biopsies

**Figure 6:**
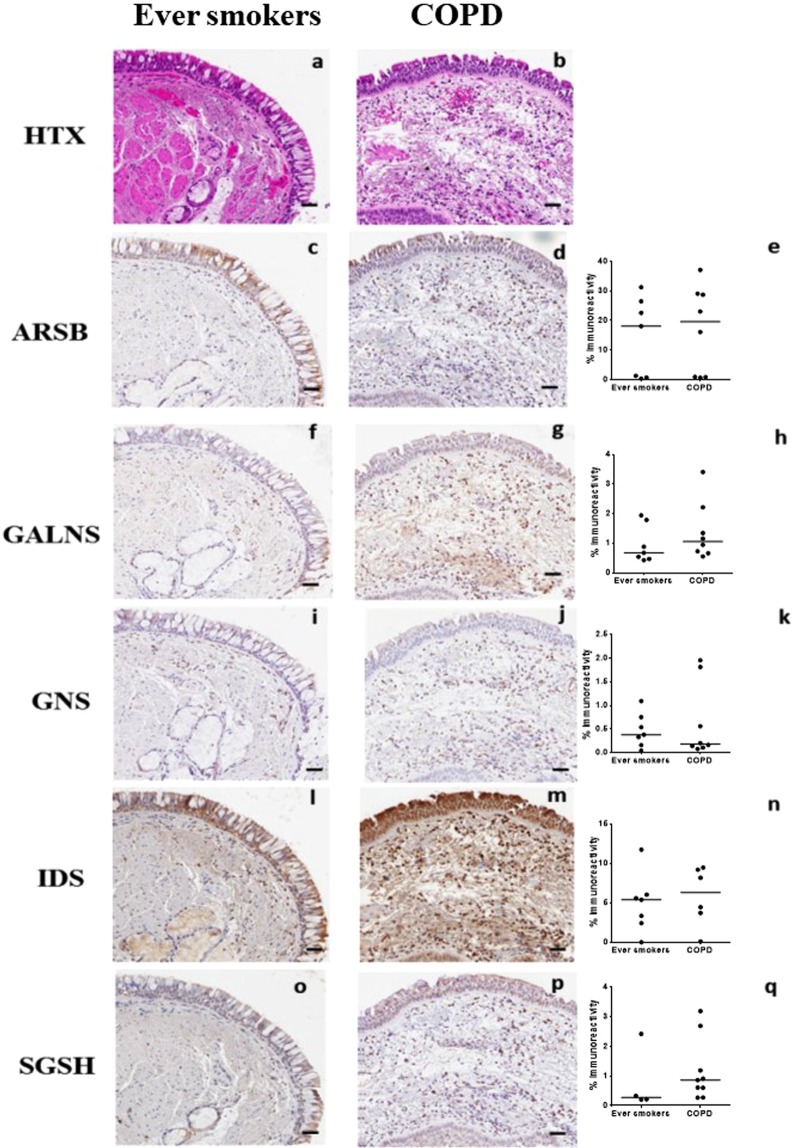
Examination of sulfatases in bronchial biopsies. Bronchial biopsies were stained for the lysosomal sulfatases. Tissue sections from ever smokers (**a**,**c**,**f**,**i**,**l**,**o**) were compared to COPD patients (**b**,**d**,**g**,**j**,**m**,**p)**. Panels **a** and **b** were stained with hematoxylin (HTX) to label cells and nuclei, whereas the remaining panels were stained with anti-ARSB (**c**,**d**), anti-GALNS (**f**,**g**), anti-GNS (**i**,**j**), anti-IDS (**l**,**m**) and anti-SGSH (**o**,**p**). All panels are representative images of ever smokers and COPD patients. Graphs **(e**,**h**,**k**,**n**,**q)** show quantifications of the different individuals, and each dot represents one subject. Scale bar equals 50 µm.

As we primarily examined cultured fibroblasts, we aim to determine if the histological pattern of lysosomal sulfatase differed in biopsies from the airways. We found that overall, there was expression of all examined lysosomal sulfatases in bronchial biopsy tissue from ever smokers and COPD patients (Fig. 6c,f,i,l,o,d,g,j,m,p, respectively). When the overall intensity was analyzed, we found that there was a non-significant trend towards increased intensity in GALNS, IDS, and SGSH (Fig. 6h,n,q). When comparing ever smokers to COPD patients, it became apparent that there was a prominent staining of cells in the lamina propria and this staining was observed in all COPD subjects. There were also differences in staining amongst the five sulfatases. For example, ARSB showed a reduced staining in epithelial cells from COPD patients compared to ever smokers (Fig. 6c,d). Furthermore, ARSB, IDS and SGSH showed prominent staining in the epithelium of ever smokers and COPD patients (Fig. 6c,d,l,m,o,p), whereas this staining of GALNS and GNS was not as readily observed (Fig. 6f,g,I,j).

### *SUMF1* mRNA variants correlate with sulfatase mRNA expression

Correlation analyses were performed between mRNA expression of *SUMF1* splice variants and lysosomal sulfatases in lung fibroblasts from ever smokers and COPD patients. *SUMF1* splice variants 1–3 (*Sv1-3*) as described previously^26^ were examined and strong significance was found between both *Sv1* and *Sv2* and the sulfatases *ARSB*, *GALNS*, and *IDS* (Table 1). Additionally, *Sv3* significantly correlated with *GNS*.

**Table 1:** Correlations between *SUMF1* mRNA variants and sulfatase mRNA expression.

	*SUMF1*			
	*SUMF1* total	Splice variant 1	Splice variant 2	Splice variant 3
**Ever smokers and COPD patients**				
*ARSB*	r = 0.16 p = 0.53	***r*** = ***0***.***86*** ***p*** < ***0***.***0001***	***r*** = ***0***.***66*** ***p*** = ***0***.***0052***	r = 0.37 p = 0.15
*GALNS*	r = −0.05 p = 0.85	***r*** = ***0***.***56*** ***p*** = ***0***.***020***	***r*** = ***0***.***64*** ***p*** = ***0***.***0071***	r = 0.24 p = 0.36
*GNS*	r = 0.41 p = 0.11	r = 0.19 p = 0.47	r = 0.32 p = 0.22	***r*** = ***0***.***50*** ***p*** = ***0***.***045***
*IDS*	r = −0.02 p = 0.94	***r*** = ***0***.***81*** ***p*** = ***0***.***0001***	***r*** = ***0***.***80*** ***p*** = ***0***.***0002***	r = 0.28 p = 0.27
*SGSH*	r = −0.06 p = 0.83	r = −0.47 p = 0.065	r = −0.34 p = 0.19	r = −0.15 p = 0.57
**COPD patients only**				
*ARSB*	r = 0.05 p = 0.88	r = ***0***.***87*** p = ***0***.***0002***	r = ***0***.***66*** p = ***0***.***017***	r = 0.28 p = 0.35
*GALNS*	r = 0.14 p = 0.64	r = ***0***.***62*** p = ***0***.***028***	r = 0.52 p = 0.071	r = 0.36 p = 0.22
*GNS*	r = ***0***.***60*** p = ***0***.***034***	r = −0.09 p = 0.76	r = 0.02 p = 0.96	r = 0.40 p = 0.18
*IDS*	r = 0.28 p = 0.35	***r*** = ***0***.***93*** ***p*** < ***0***.***0001***	***r*** = ***0***.***66*** ***p*** = ***0***.***017***	r = 0.42 p = 0.16
*SGSH*	r = −0.06 p = 0.86	r = −0.50 p = 0.085	r = −0.30 p = 0.32	r = −0.15 p = 0.63

Spearman correlation test was performed to identify correlations between *SUMF1* mRNA and mRNA from the various examined sulfatases. Correlations were performed on ever smoking and COPD samples as well as on COPD patients only. *Bold italics* indicate statistical significance.

When only COPD subjects were examined, *Sv1* still correlated with *ARSB*, *GALNS* and *IDS*. Interestingly, only *ARSB* and *IDS* correlated with *Sv2* and none of the sulfatases correlated with *Sv3*. When taking into account the total expression of *SUMF1* (including all splice variants) there was only a correlation with *GNS* in COPD patients.

## Discussion

In our study, we show that sulfatase expression, both in mRNA and protein, as well as sulfatase activity was altered in lung fibroblasts and lung tissue from patients with COPD. Recently, our group has found that changes of the sulfatase modifying factor, *SUMF1*, was associated with COPD^26^. Thus, we explored if sulfatases, which are directly activated by SUMF1, were also affected in COPD patients. The role of sulfatases in the body has been the topic of several studies in recent years. Sulfatases are best known for their role in disease, most notably mucopolysaccharidosis where one or more sulfatases are non-functional^5,6^. Despite the advancements in our knowledge of sulfatases, there are still a plethora of unanswered questions.

Although COPD is a heterogeneous disease, we found altered sulfatase patterns in our subjects. We found that mRNA expression of the five examined lysosomal sulfatases was increased in COPD patients (Fig. 1). Interestingly, when examining smoking status in our COPD subjects (all ever smokers represented in Fig. 1 were former smokers), we found that there was some clustering based on current or former smokers (Fig. 1A,C). This finding suggests that smoking status may influence the expression of particular sulfatases. Additional investigation into the effect of smoking on sulfatase expression would be interesting for future experiments.

In several patients, we also found increases in the activity of one or more sulfatases (Fig. 2). This increase in sulfatase activity in COPD could point to overactive sulfatases or even increases in the activity of the modifying factor SUMF1. In contrast, protein expression of lysosomal sulfatases in COPD patients showed an overall decrease (Figs 3 and S1). When examining intracellular localization of the sulfatases in cultured primary fibroblasts, we found that some sulfatases (ARSB, IDS, SGSH and GNS) showed mis-localization from their reported lysosomal compartments (Fig. 4). Perhaps the mis-localization of these sulfatases led to the protein decrease (due to increased protein degradation) seen by western blotting. These findings, taken together, may suggest a yet undetermined feedback loop for sulfatases that is present in COPD patients. Additionally, fibroblasts are known to produce many of the factors responsible for shaping the extracellular matrix of the cell^27^. Many lysosomal sulfatases can be exported to the intercellular space/extracellular matrix and later internalized^28^. Due to this reinternalization, there is the possibility that the decrease in intracellular sulfatase protein levels could be partially due to an increase in exported proteins. An alternative possibility is that the mis-localization of sulfatases seen in fibroblasts by immunofluorescence may be due to shuttling through the anterograde transport system or even modifications of re-internalized proteins as these are currently indistinguishable from newly synthesized sulfatases. Though in this current study we did not examine re-internalization of the enzymes via retrograde transport. Both of these topics would, thus, be of interest to better decipher the changes to lysosomal sulfatase levels in COPD patients. Furthermore, the lack of discrepancy between mRNA and protein levels could also be due to other mechanisms, such as, for example, post-translational modifications, regulation by RNA binding proteins, regulation by small RNA species or difference in protein half-lives.

Although our immunofluorescence staining indeed indicated that not all examined lysosomal sulfatases were correctly localized in our fibroblast cultures, they are only a snapshot of what is happening in the cell. To further delve into the localization of sulfatases such as SGSH and ARSB, for example, it would be beneficial to stain for more marker proteins, such as endosomal components, where sulfatases may be found en route to anterograde or retrograde transport. Ideally, live cell imaging and *in vivo* examination of sulfatase trafficking would answer many questions, such as if there are potential blocks in the endocytic system in COPD patients or whether sulfatases in COPD patients are more readily exported to the intracellular space/extracellular matrix, which may explain a decrease in their protein expression within the cellular fraction.

The five lysosomal sulfatases analyzed in this study are involved in modifying the sulfation pattern of the glycosaminoglycans chondroitin/dermatan sulfate and heparan sulfate in the extra cellular matrix. ARSB, GALNS and IDS target the sulfate groups of chondroitin/dermatan sulfate chains, and GNS, IDS and SGSH target the sulfate groups of heparan sulfate chains^5^. Though, in this study the altered sulfatase expression does not differ between sulfatases targeting chondroitin/dermatan^5,6^ sulfate versus heparan sulfate. This suggests an overall effect of the sulfation pattern of the glycosaminoglycan chains in COPD, similar to what has been shown, for example, in idiopathic pulmonary fibrosis^29^. This may result in an altered extracellular matrix remodeling balance related to the emphysema formation in COPD.

It appears in the hematoxylin and eosin (HTX) staining that there is an increase in cells in the lamina propria of COPD patients (Fig. 6) and these cells also appear to contribute to the increased staining of sulfatases. Perhaps these cells, seen in whole tissue by IHC, are fibroblasts or another sulfatase-rich cell type and this increased staining might suggest an increase in the production of exported proteins. Marker proteins would be helpful to aide in the further identification of these cells by IHC analysis and what their potential role is in COPD. We should also keep in mind that we are investigating former and current smoking subjects, both healthy and COPD patients. This study does not include never smoking COPD patients, which might have a totally different pathological picture, but this aspect is yet to be determined.

Strengthening the important role of SUMF1 in relation to sulfatases, we found that gene expression of *SUMF1* variants correlated with the gene expression of several of the lysosomal sulfatases Table 1). *Sv1* represents the entire transcript whereas *Sv2* and *Sv3* are lacking exon 3 and 8, respectively. Our correlations suggest that *Sv3* acts in a different manner than the other variants as only *GNS* was seen to correlate when ever smokers and COPD patients were collectively taken into account. The entire mRNA transcript (*Sv1*) strongly correlated with 3 of the 5 lysosomal sulfatases, *ARSB*, *IDS*, and *GALNS*. Furthermore, IDS activity levels were also elevated in all COPD patients that we examined. These findings strengthen the relationship between SUMF1 and its downstream targets, the sulfatases. One could think that either there is a direct feedback loop so that when more sulfatase mRNA is produced, then more of the regulator, SUMF1, is also produced putting the whole system into a constant state of checks and regulation. Further work in the direct relationship between SUMF1 in the lung and lysosomal sulfatases would further help to uncover the important role of sulfatases in the lung.

As with any study, we were faced with a number of limitations. One such limitation of this study was the inability to test the activity of all 17 human sulfatases. Currently, sulfatase activity assays are only clinically available on the five described lysosomal sulfatases as they are often mis-regulated in lysosomal storage disorders^5,6,30–36^. Thus, we were unable to test the majority of sulfatases for changes in activity. It would have been especially interesting to also examine the ARSJ as it was most highly expressed transcript in lung tissue (Fig. S2). Furthermore, it would be of interest to see if the other described lysosomal sulfatases: ARSA, -G, and –K, behave in a similar manner to the five lysosomal sulfatases that we have currently examined. Another limitation was the number of never smokers and ever smokers used in this study. Recruitment of these subjects, especially age matched, for bronchoscopy proved difficult. Finally, our bronchoscopy samples, were obtained from the central airway. COPD is a disease that strongly manifests in the peripheral airways, so material from the periphery would have been interesting to analyze in comparison. As mentioned previously, glycosaminoglycan accumulation was seen in *Sumf1*−/− mice in the alveoli^16^. Perhaps in humans the effects of sulfatases in the lung are also more pronounced in the distal airways and the alveoli.

To our knowledge, this is the first study exploring sulfatases in-depth in the human lung. We have investigated never smokers, ever smokers and COPD patients and have shown altered expression, localization, and activity of five lysosomal sulfatases. In each of these cases, we have found that COPD patients differed from our control groups, indicating that an altered pattern of sulfatases may play a role in the pathophysiology of the disease. We hope that new advances into the study of sulfatases will continue to piece together this very interesting puzzle, which in turn could lead to improve treatment of COPD.

## Methods

### Patients

All subjects performed spirometry and COPD was defined according to the GOLD criteria: Force Exhaled Volume in 1 sec/Forced Vital Capacity (FEV1/FVC) < 0.7^4^. Lung function measurements were performed according to the manufacturer’s instructions and European Respiratory Society/American Thorax Society guidelines/recommendations^37–39^. Twenty ever smoking (current or former) COPD patients, 8 ever smokers (current or former) with normal spirometry and 6 never smokers without any respiratory disease and normal spirometry were included. Patients had no other significant cardiorespiratory disease, no lower respiratory tract infection within three weeks prior to bronchoscopy and ever smokers had at least 15 pack years. Detailed patient characteristics can be found in Table 2. The methods were carried out in accordance with the relevant guidelines and regulations. All patients signed the written informed consent and the study was approved by the Regional Ethical Review Board in Lund, Sweden (431/2008).

**Table 2:** Characteristics of study subjects.

	Never smokers, n = 6	Ever smokers, n = 8	COPD, n = 20
Gender (female/male), n	4/2	3/5	8/12
Smoking status (current/former), n	0	1/7	11/9
Pack years*	0	33 (24–38)	43 (35–49)
FEV1, L	3.4 (3.2–3.6)	3.3 (2.6–3.4)	1.7 (1.5–2.1)
FEV1, % of predicted	114 (95–139)	95 (93–98)	54 (49–70)
FEV1/FVC	0.84 (0.81–0.88)	0.78 (0.74–0.81)	0.50 (0.44–0.60)

^*^A pack-year is defined as smoking 20 cigarettes (=one pack) per day for a year.

Pack years, FEV1, FEV1 (%p) and FEV1/FVC are given as median with interquartile range (25–75%).

### Bronchoscopy Sampling and Cell culture

The bronchoscopy procedure was performed as previously described by Tufvesson, *et al*.^40^. In summary, the bronchoscopies were performed under local anesthetics and were done in accordance with clinical routines. A flexible bronchoscope (Olympus IT60, Tokyo, Japan) with a working channel of 2.6 mm was used in combination with biopsy forceps (Olympus FB211D). Bronchial biopsies were taken from the central airways in segmental and subsegmental carina, and lung fibroblasts were isolated via outgrowth from these biopsies. Cells were grown in Dulbecco’s modified eagle medium (DMEM; (SigmaAldrich, St Louis, MO) supplemented with 10% FetalClone^TM^ III Serum (Fischer Scientific, Waltham, MA), 1% amphotericin, 0.5% gentamicin and 0.1% L-Glutamine (all from SigmaAldrich) and were cultured for 3–4 passages before harvesting. Cells were harvested via addition of trypsin (SigmaAldrich) to the cells, then rinsing with 1xPhosphate buffered saline (PBS; SigmaAldrich) and spinning the cells at 500 x g for 5 minutes to pellet them. Pelleted cells were then used for RNA extraction.

For cells used in immunofluorescence staining, fibroblasts were seeded into 4 well chamber slides (SigmaAldrich) at a density of 20,000 cells per well and allowed to grow to semi-confluency. Media was then removed and cells were fixed with 4% paraformaldehyde (SigmaAldrich) and subsequently washed 3x with PBS and stored in PBS at 4 °C until staining.

Bronchoscopy was performed on all subjects, but for ever smokers with normal spirometry, biopsy fibroblasts from only 4 out of 8 subjects grew out *ex vivo*, yielding viable fibroblast cultures.

### RNA extraction and qPCR analysis

Cells pelleted, as mentioned above, were lysed using RNeasy Lysis Buffer (Qiagen, Hilden, Germany) with 1% β-mercaptoethanol. RNA was prepared using an RNeasy Mini kit according to manufacturer’s instructions (from Qiagen GmbH, Hilden, Germany). cDNA was synthesized using iScript™ cDNA Synthesis Kit from Bio-Rad Laboratories (Hercules, CA) following the manufacturer’s instructions and synthesis program: 5 min 25 °C, 30 min 42 °C and 5 min 85 °C. Semi-quantitative real-time PCR (qPCR) was performed using 1 ng cDNA per well using iTaq™ SYBR Green Supermix with ROX from Bio-Rad Laboratories on an Applied Biosystems 7900 thermocycler (ThermoFisher, Waltham, MA) as described previously^26^. All primers, forward and reverse (Invitrogen), were used at a final concentration of 300 nM. Primers against the lysosomal sulfatases *ARSB*, *GALNS*, *GNS*, *IDS*, and *SGSH* were examined in lung fibroblasts. The qPCR program was as follows: 95 °C 10 min, then 45 cycles: 15 sec at 95 °C, 30 sec at 60 °C, 30 sec at 74 °C, followed by a melt curve. The cycle threshold value (Ct) was determined by the Applied Biosystems software. Data are depicted as 2^−∆Ct^_._

All 17 reported human sulfatases were examined in RNA from the Human Total Master Panel II Lot# 1505145 A (TakaraBio-Clontech, Saint-Germain-en-Laye, France).

Expression of all mRNA was normalized against the average expression of the two reference genes beta-actin *(β-actin)* and glyceraldehyde-3-phosphate dehydrogenase (*GAPDH)*^26^. For all primer sequences, see Table S1.

### Sulfatase Activity Assays

All sulfatase activity assays were performed according to clinical routine at Clinical Chemistry, Sahlgrenska Hospital (Gothenburg, Sweden) on lung fibroblasts isolated from COPD patients. The clinically used normal ranges for healthy subjects were used as the respective reference ranges. The method was based on previously published assays for ARSB, GALNS, GNS, IDS, and SGSH, and measurement of beta-galactosidase was used as a control to ensure correct estimation of sulfatase activity^31–36^.

### Western blotting

Cultured lung fibroblasts were manually lysed using NP-40 cell lysis buffer (Invitrogen, Bender MedSystems GmbH, Vienna, Austria). Briefly, cell culture media was removed from confluent fibroblasts. NP-40 lysis buffer was added to cells and incubated for 5 minutes on ice. Cells were then scraped, lysate was collected and spun at 10 000 × g for 10 min at 4 °C. The supernatant was removed and used to determine total protein using a BCA assay (ThermoFisher). Laemmli sample buffer (4X, Bio-Rad) was added to 20 μg of total protein to make the final Laemmli concentration 1x and the sample was placed at 95 °C for 5 minutes. Samples were allowed to cool to room temperature before loading. Immunoblotting was performed according to the Bio-Rad mini PROTEAN system protocols (Bio-Rad), and samples were run on 4–20% Mini-PROTEAN TGX stain free protein gels in 1x Tris/Glycine/SDS (Bio-Rad). Proteins were then transferred to PVDF membranes (Bio-Rad; PVDF transfer packages) using a Bio-Rad Trans-Blot^®^ Turbo™ Transfer System. After transfer, membranes were imaged to ensure transfer of protein from the gel and membrane were then rinsed once in distilled water before transferring into blocking solution (3.5% milk in 1xTris Buffered Saline with 0.1% Tween20 (TBST)). Membranes were blocked for 1 h at room temperature. After 1 h the blocking solution was discarded and primary antibody (for concentrations used see Table S2) in blocking solution was added and the gels were allowed to incubate with shaking at 4 °C overnight. Primary antibody was discarded and blots were washed 3x in 1x TBST before adding secondary antibody coupled to HRP (Table S2) in blocking solution and left to incubate 1.5 h at room temperature. Finally, the secondary antibody solution was discarded and blots were washed 3x in 1x TBST and 1x in MilliQ water. Blots were observed using ECL chemiluminescence (ThermoFisher) and imaged on a Bio-Rad ChemiDoc Imaging system (Bio-Rad).

### Immunofluorescence and Microscopy

Fibroblasts grown in chambered slides, as mentioned above, were used for staining. Cells were permeabilized with PBST (1% Tween20 in PBS) and blocked with blocking buffer (2% Normal Goat Serum (NGS) for all sulfatases, except IDS which was blocked in 2% Normal Donkey Serum (NDS), both from Abcam, Cambridge, UK, in PBST). Cells were incubated with primary antibodies in blocking buffer, washed with PBST, and thereafter incubated with secondary antibodies in blocking buffer. Cells were washed with PBST, followed by a PBS wash and a final deionized water wash. Anti-fading mounting solution containing DAPI (Invitrogen, Massachusetts, USA) and a coverslip was then added to each slide. Slides were stored at 4 °C until analyzed. Images were taken using an Olympus DP80 camera mounted on a Nikon Eclipse 80i fluorescence microscope with a 40x Plan-Apochromat objective. The software used for imaging was CellSens Dimension (CellSens, The Netherlands). Image processing was performed using ImageJ (NIH, Maryland, USA).

### Immunohistochemistry

At the bronchoscopy, bronchial biopsies from each patient were immediately immersed into 4% buffered paraformaldehyde. Tissues were processed and dehydrated, and paraffin-embedded tissue blocks were prepared for histological analysis. Sections from 5–6 biopsies from each ever smoking individual or COPD patient were placed on the same slide to ensure a similar staining protocol for all biopsies.

Immunostaining was performed on 4 µm tissue sections using an automated slide staining robot (Autostainer Plus, Dako). After deparaffinization, antigen retrieval was performed using PT link (Dako) by heating the tissue sections in a target retrieval solution buffer at pH 6.0 (Dako). The immunohistochemistry protocol incorporated anti-mouse/rabbit polymer HRP as a secondary antibody from Envision^TM^ Flex (K8010, Dako) Peroxidase/DAB Rabbit/Mouse detection systems. A single staining protocol was used for staining ARSB^+^, GALNS^+^, GNS^+^, IDS^+^, and SGSH^+^ cells using DAB as detection chromogen. Details of primary and secondary antibodies are presented in Table S2. Each slide was counterstained with Mayer’s haematoxylin. Specificity was confirmed by omitting the primary antibody, and a subsequent lack of staining.

All stained slides were digitally scanned using an automated Scanscope digital slide scanner (Aperio Technologies, CA, USA) to obtain high-resolution digital images of stained bronchial biopsies. Immunoreactivity was calculated by dividing the strong positive pixels for a marker with the total positive and negative pixels obtained by using Imagescope software (Aperio Technologies) in a selected tissue area.

### Antibodies

For western blotting (WB), immunofluorescence (IF) staining, and immunohistochemistry (IHC) antibodies against sulfatases and various marker proteins against cellular organelles were used. Antibodies and concentrations used in this study can be found in Table S2.

### Statistics

Descriptive statistics are presented as median (interquartile range (IQR)). P < 0.05 was considered significant.

The differences in gene expression and protein expression in fibroblasts amongst never smokers, ever smokers, and COPD patients were analyzed using Kruskal-Wallis test including Dunn’s Multiple Comparison Post Test. Comparison of sulfatase immunohistochemistry staining was analyzed using a non-parametric t-test (Mann-Whitney test). Spearman correlation test was performed to determine the relationship between *SUMF1* and sulfatase mRNA levels. Statistical analysis was performed using Graph Pad Prism 6 (Graph Pad software Inc. San Diego, CA).

## Supplementary information

Supplemental Materials
