# Supplementary material for: Expression, activity and localization of lysosomal sulfatases in Chronic Obstructive Pulmonary Disease
Source: Sci Rep. 2019 Feb 13;9:1991. doi: 10.1038/s41598-018-37958-w (PMC6374378; doi:10.1038/s41598-018-37958-w)
Supplement: Supplementary file 1 — Supplemental Materials [file 41598_2018_37958_MOESM1_ESM.pdf]

Expression, activity and localization of lysosomal sulfatases in Chronic Obstructive Pulmonary Disease

Julie Weidner[1], Prajakta Jogdand[2], Linnea Jarenbäck[1], Ida Åberg[1], Dalja Helihel[1], Jaro Ankerst[1], Gunilla Westergren-Thorsson[3], Leif Bjermer[1], Jonas S Erjefält[2] & Ellen Tufvesson[1*]

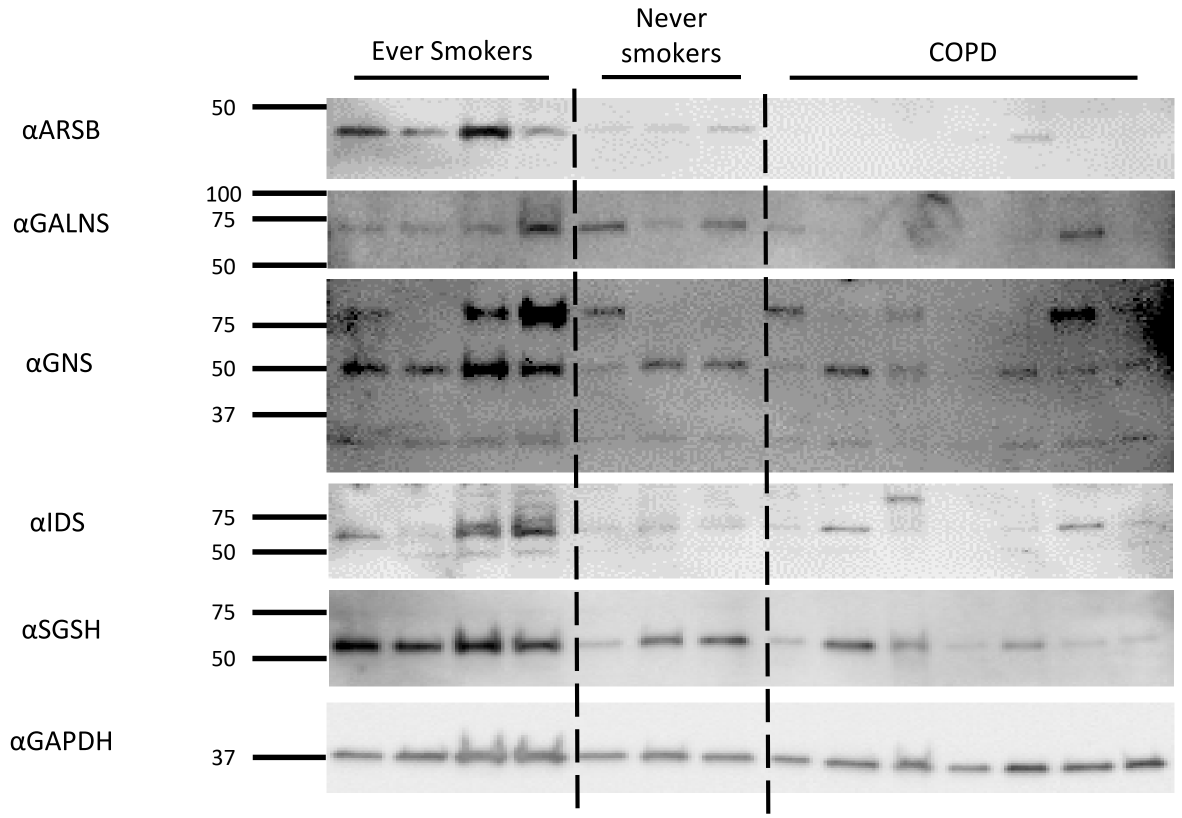

Fig S1: Western Blot analysis of sulfatases in lung fibroblasts

Whole cell fibroblast lysates from ever smokers, never smokers, and COPD patients were probed for different lysosomal sulfatases (ARSB, GALNS, GNS, IDS, and SGSH) and GAPDH as control. Each lane on the blot represents an individual subject.

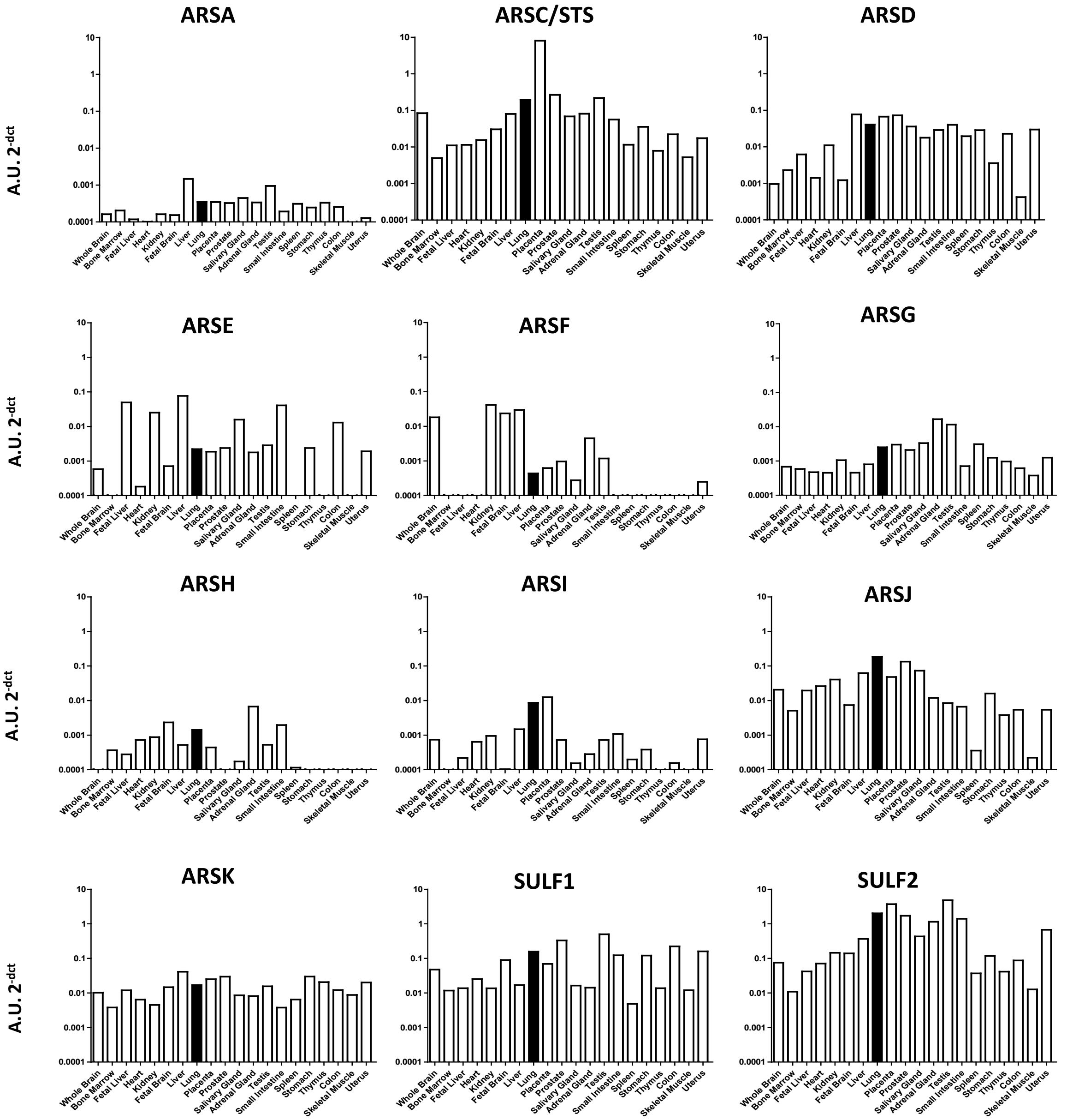

Fig S2: Examination of remaining human sulfatases

The remaining 12 known human sulfatases (ARSA, ARSC-K, SULF1 and SULF2) were examined in RNA from 20 different tissues using the Human Total Masterpanel II RNA Kit. Whole lung tissue is indicated by the black bar. Data are depicted as $2^{-\Delta Ct}$ showing the expression of all specific mRNA normalized against the average expression of the reference genes *β-actin* and *GAPDH*. A.U. = arbitrary units

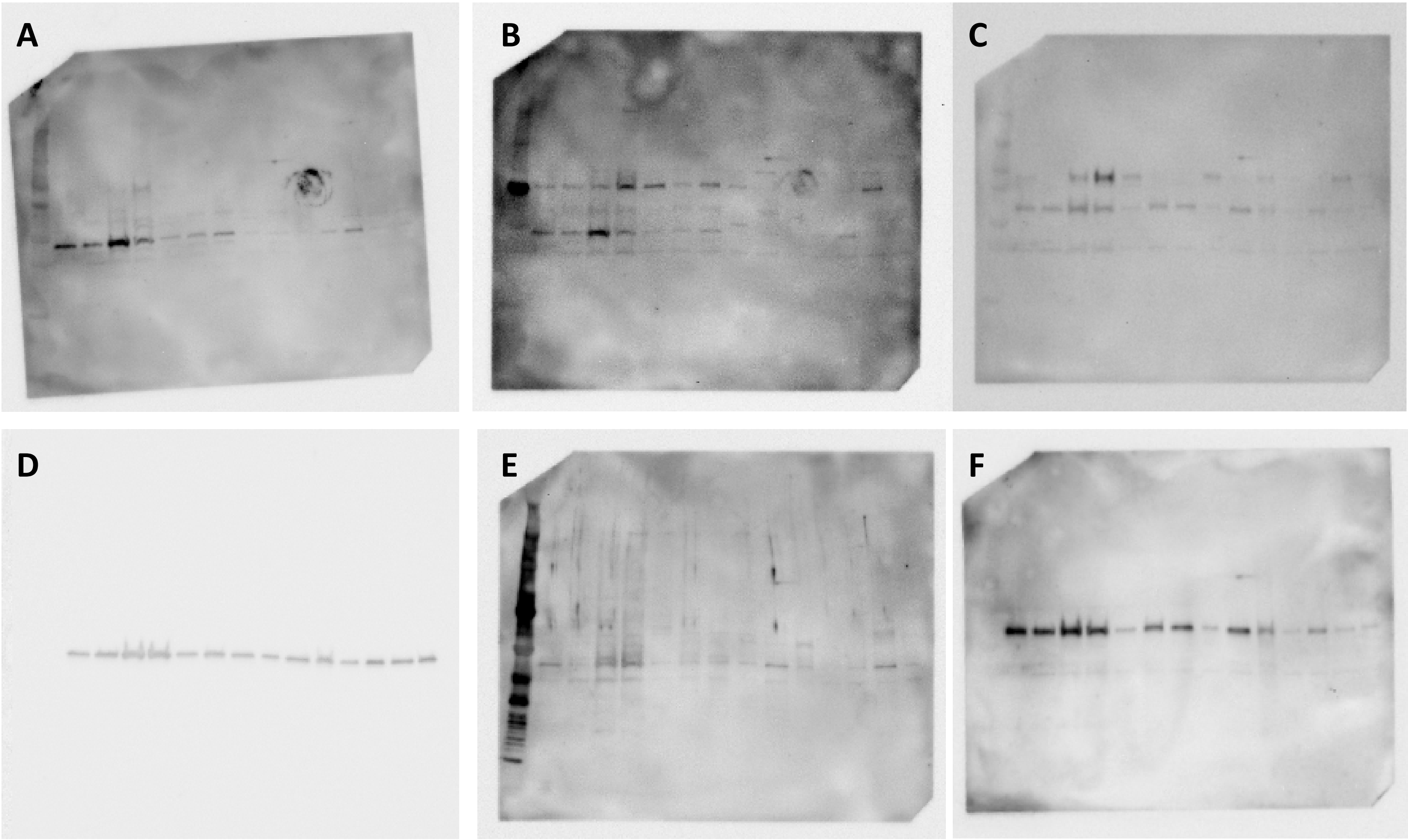

Fig S3: Full Western Blots used

Full sized blots used in the creation of Figure S1. The order is the same as represented in Fig S1. The same blot was stripped and reprobed with a new antibody (listed in table S2). (**A**) ARSB (**B**) GALNS (**C**) GNS (**D**) GAPDH (**E**) IDS (**F**) SGSH.

Table S1. Primers used in this study

Primer name	Sequence
β-Actin	5’ AGC ACA GAG CCT CGC CTT T
	3’ GGA ATC CTT CTG ACC CAT GC
GAPDH	5’ GAA GGT GAA GGT CGG AGT CA
	3’ TGG AGG ATG GTG ATG GGA TT
ARSA	5’ TCA GAT CTC CGC TCG AGA AT
	3’ CAG CAG GCT CTT TCC GAT AC
ARSB	5’ CGC CGA GGA TTT GAT ACC TA
	3’ ACA GAG GCT TCT CTG GTG GA
ARSC/STS	5’ GGA AGG CCT TTT TCT TCA CC
	3’ AGG GTC TGG GTG TGT CTG TC
ARSD	5’TCC TTT CTT TGC TGC ATG TG
	3’CCC TAA CTG GCT GTG TCC AT
ARSE	5’ ACT GGT AGC AGG GAA GCT CA
	3’ TAA GGG GTG TCG TTC TTT GG
ARSF	5’ GGA CGA CAG TGG GTC AGT TT
	3’ GTG TCA GGG GTG TGG ACT CT
ARSG	5’ AAA CTC GTC AAG GGG GAA GT
	3’ GGG CTA CCA CAG TTG GAA AA
ARSH	5’ ATA TGG CAG GTA TGG GGA CA
	3’ CAT TCC TTT GCC ACC TTT GT
ARSI	5’ TGC AAG CTT CGA TCC TTT TT
	3’ ATT CCC ACC CAC TCC TAT CC
ARSJ	5’ TCC AGA GAC TTC TGC CAC CT
	3’ CAC AGC AGT GGA ACT CAG GA
ARSK	5’ CTC CTC AGA CCA TGG AGA GC
	3’ CTC AGG TTC TGA GGC AGA GG
GALNS	5’ ATG GAC CTC TTC ACC ACC AG
	3’ GAG TTG GTC CAG GTC CAG AA
GNS	5’ GTT TAA GGG ACC CAC TGC AA
	3’ CTT TGC ATG AGA GGG AGA GC
IDS	5’ CGT CAG CCA GTC CTT TCT TC
	3’ GAC CAT ACG GCA CAC TGA TG
SGSH	5’ CAT GGG TCG TAT CCC AGA CT
	3’ CAC CAG TCC AAC TCC TTG GT
SULF1	5’ AAG GTT AAT CAG CCC CGT CT
	3’ ACC AAG AAC CCG TCA CTT TG
SULF2	5’ GAG CTG CAA GGG TTA CAA GC
	3’ CTT CCC ACA GTT GTC CCA GT

Table S2. Antibodies used in this Study

Primary Antibody	Application	Dilution	Host/ Isotype	Manufacturer	Secondary Antibodies, Invitrogen (IF, WB)
**SGSH**	IF, WB , IHC	1:1000 (IHC) 1:750 (WB) 1:100 (IF)	Rabbit, polyclonal IgG (WB, IHC) Rabbit, Monoclonal (IF)	Abcam ab96029 (WB, IHC) ab200346 (IF)	goat anti-rabbit Alexa488 (IF) goat anti-rabbit HRP (WB)
**GALNS**	IF, WB , IHC	1:200 (IHC) 1:750 (WB) 1:100 (IF)	Rabbit, polyclonal IgG	Abcam ab97913 (IHC, WB, IF)	goat anti-rabbit Alexa488 (IF) goat anti-rabbit HRP (WB)
**GNS**	IF, IHC, WB	1:400 (IHC) 1:750 (WB) 1:100 (IF)	Rabbit, polyclonal IgG	Abcam ab111441 (IHC, WB) R&D systems AF2484 (IF)	goat anti-rabbit Alexa488 (IF) goat anti-rabbit HRP (WB)
**ARSB**	IF, WB , IHC	1:200 (IHC) 1:750 (WB) 1:100 (IF)	Rabbit, polyclonal IgG	Atlas Antibodies HPA037771 (IHC, WB, IF)	goat anti-rabbit Alexa488 (IF) goat anti-rabbit HRP (WB)
**IDS**	IF, WB , IHC	1:1200 (IHC) 1:1000 (WB) 1:200 (IF)	Goat, polyclonal IgG	Abcam ab85701 (IHC, WB, IF)	donkey anti-goat Alexa555 (IF) donkey anti-goat HRP (WB)
**LAMP1**	IF	1:250	Mouse monoclonal	Abcam ab25630	rabbit anti-mouse Alexa 647
**Golgin97**	IF	1:250	Mouse monoclonal	Thermo/life technologies A21270	rabbit anti-mouse Alexa 647
**GAPDH**	WB	1:3000	Mouse monoclonal	Santa Cruz sc-47724	goat anti-mouse HRP
**DAPI**	IF	---	----	Invitrogen ProLong Gold Antifade reagent with DAPI (P36935)	
